# Vitamin C for Cardiac Protection during Percutaneous Coronary Intervention: A Systematic Review of Randomized Controlled Trials

**DOI:** 10.3390/nu12082199

**Published:** 2020-07-23

**Authors:** Sher Ali Khan, Sandipan Bhattacharjee, Muhammad Owais Abdul Ghani, Rachel Walden, Qin M. Chen

**Affiliations:** 1 Department of Pharmacy Practice and Science, College of Pharmacy, University of Arizona, 1295 N. Martin Ave, Tucson, AZ 85721, USA; sheralikhan6161@gmail.com (S.A.K.); bhattacharjee@pharmacy.arizona.edu (S.B.); 2 Department of Cardiac Surgery, Vanderbilt University, Nashville, TN 37203, USA; owais.a.gh@gmail.com; 3 Annette and Irwin Eskind Family Biomedical Library, Jean & Alexander Heard Libraries, Vanderbilt University, Nashville, TN 37203, USA; Rachel.l.walden@vanderbilt.edu

**Keywords:** Vitamin C (VC), periprocedural myocardial injury (PMI), troponin, CK-MB, left ventricular ejection fraction (LVEF), reactive oxygen species (ROS)

## Abstract

Percutaneous coronary intervention (PCI) is the preferred treatment for acute coronary syndrome (ACS) secondary to atherosclerotic coronary artery disease. This nonsurgical procedure is also used for selective patients with stable angina. Although the procedure is essential for restoring blood flow, reperfusion can increase oxidative stress as a side effect. We address whether intravenous infusion of vitamin C (VC) prior to PCI provides a benefit for cardioprotection. A total of eight randomized controlled trials (RCT) reported in the literature were selected from 371 publications through systematic literature searches in six electronic databases. The data of VC effect on cardiac injury biomarkers and cardiac function were extracted from these trials adding up to a total of 1185 patients. VC administration reduced cardiac injury as measured by troponin and CK-MB elevations, along with increased antioxidant reservoir, reduced reactive oxygen species (ROS) and decreased inflammatory markers. Improvement of the left ventricular ejection fraction (LVEF) and telediastolic left ventricular volume (TLVV) showed a trend but inconclusive association with VC. Intravenous infusion of VC before PCI may serve as an effective method for cardioprotection against reperfusion injury.

## 1. Introduction

Cardiovascular disease (CVD) is the number one cause of death worldwide [1]. CVD claimed 859,125 lives in 2017 in the United States [2] or 17.9 million in 2016 worldwide [1]. Among all forms of CVD, acute coronary syndrome (ACS) accounts for 40–50% of CVD deaths and is a leading cause of mortality and morbidity [3,4]. Common presentations of ACS include unstable angina, ST-elevation myocardial infarction (STEMI), and non-ST elevation myocardial infarction (NSTEMI) [5,6]. The advancement of medical science has led to effective management of ACS, mostly with percutaneous coronary intervention (PCI) and adjuvant pharmacological therapy [6,7,8,9,10,11,12]. About 90% of STEMI patients or 50% of NSTEMI patients are treated with PCI [13]. Despite the improvement in PCI technology, there is a possibility that patients can suffer from complications, including a bleeding event, hematoma, re-infarction, cardiogenic shock, heart failure, and death in the worst-case scenario [14,15,16,17,18].

The PCI procedure results in a periprocedural myocardial injury (PMI) in 5–30% of the patients [11,19,20]. Such injury is measurable by biomarkers of myocardial cell death, i.e., elevation of cardiac troponin I (cTnI) and creatinine phosphokinase MB isoenzyme (CK-MB) in the circulation [19,21]. While myocardial revascularization is essential for relieving symptoms and preventing death, reperfusion can cause escalation of oxidative stress on top of ischemia and is estimated to account for 40% of the final infarct size [22,23]. Reperfusion injury can lead to fatal arrhythmias and re-infarction [3,24]. Reactive oxygen species (ROS) are detectable in the blood circulation in higher levels within the first few minutes of reperfusion [25,26,27,28,29,30,31]. ROS produced during reperfusion are believed to be an important cause of cell death and procedure-related complications [23,32].

Clinical studies have confirmed that antioxidant supplements are indeed effective in reducing ROS measured in the serum [33,34]. Vitamin C (ascorbic acid or ascorbate, VC) is a water soluble molecule that can be administered orally or via an intravenous route [35]. VC is a classic antioxidant that has a proven record for scavenging ROS, but whether it plays a role in reducing reperfusion injury has been a matter of debate [36,37,38,39,40,41,42,43]. In studies with experimental animals, administration of antioxidant agents significantly reduces ROS, appears to lower apoptotic load, and improves the outcome of reperfusion [28]. The biologic plausibility of the cardioprotective effect of VC has been shown in clinical trials where VC reduced ROS when administered prior to PCI [33,34,42,43]. However, the outcomes are conflicting, with some studies showing reduction of the biomarkers of myocardial injury cTnI and CK-MB [39,41], whereas others fail to show a benefit [29,44]. Given these inconsistences, it is prudent to address the question whether or not VC administration is benefits following PCI.

Here we address whether addition of VC to the standard PCI protocol offers protection against reperfusion related myocardial injury using a systematic review approach. Three systematic reviews and meta-analysis have determined the effect of VC following cardiac surgery, but none have been summarized the effect following PCI [45,46,47,48]. Given the fact that PCI is increasingly common for the treatment of ACS, and in selective patients for the treatment of stable angina, a systematic review of VC treatment for PCI patients can provide needed information to guide its use during clinical practice. Our primary measures include biomarkers of myocardial injury, cardiac function, and vascular perfusion indices. The potential benefit was traced to the effect of VC on the levels of reactive oxygen species (ROS) and inflammatory mediators.

## 2. Materials and Methods

The Preferred Reporting Items for Systematic Reviews (PRISMA) guideline [49] was followed for this systematic literature review using an a-priori inclusion and exclusion criteria.

### 2.1. Inclusion and Exclusion Criteria

The a priori inclusion criteria were: (1) randomized controlled trials (RCTs) assessing the effect of VC in patients greater than 18 years old who underwent PCI. (2) VC was administered within 24 h before or during PCI, (3) the control group received either placebo or standard care, (4) published and unpublished RCTs, (5) RCTs published in any language, (6) RCTs published from inception of respective databases to 18 February 2020. The excluded reports did not meet the inclusion criteria. We considered the following endpoints: myocardial injury (troponin, CK-MB), cardiac contractility (left ventricular ejection fraction, LVEF and telediastolic left ventricular volume, TLVV), restenosis of the treated coronary artery, reactive oxygen species (ROS), inflammatory mediators or markers, and vascular endothelial dysfunction.

### 2.2. Literature Search

**Figure 1:**
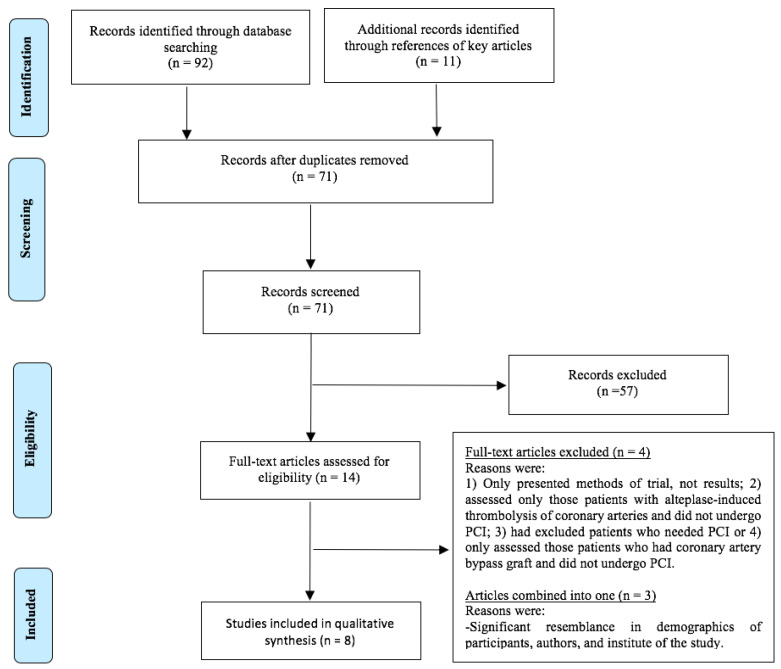
Preferred Reporting Items for Systematic Reviews and Meta-Analyses (PRISMA) Flow Diagram. The numbers document the literature search results.

The PRISMA flowchart summary is shown in Figure 1. The librarian of Vanderbilt University (Rachel Walden) was involved in developing the literature search strategies for the different electronic databases. Search for the appropriate electronic databases by primary (Sher Ali Khan) or secondary reviewer (Muhammad Owais Abdul Ghani) was performed independently in the first stage and with the input from the librarian in the second phase. No major discrepancy was noted among the two independent reviewers in shortlisted trials and quality assessment. Minor discrepancy was noted in extracted data, which was resolved with discussion reaching mutual agreement.

The search strategy used a combination of keywords and subject headings to find studies discussing the use of VC in percutaneous coronary intervention. The following terms were used to create the search strategy: ascorbic acid, ascorbicum, l-ascorbic acid, hybrin, ascorbate, vitamin C, magnorbin, angioplasty, percutaneous coronary intervention, percutaneous coronary revascularization, myocardial and coronary reperfusion, and myocardial reperfusion injury (see Supplementary Material, which presents full search strategies). Search strategy was created for PubMed using both keywords and medical subject headings and then translated to use in Embase (Ovid), Web of Science (Clarivate Analytics), Cumulative Index to Nursing and Allied Health Literature (CINAHL), Cochrane Library (Wiley) and Clinicaltrials.gov from the inception of the database till 18th February 2020. The resulting reports were screened using Raayan web application. A manual search of full content of key references was also performed. The duplicate records were removed electronically followed by careful checking.

### 2.3. Quality Assessment of Included Trials

The revised Cochrane risk of bias tool for randomized trials (RoB2) was applied to assess the risk of bias for each included trial [50]. The following domains were evaluated: random sequence generation, allocation concealment, blinding of patients and personnel, blinding of outcome assessment and incomplete outcome data. Each domain was assigned with a low, unclear or high risk of bias score.

## 3. Results

### 3.1. Characteristics of the Trials

In total 103 publications were found in which 92 were revealed with electronic search of databases and 11 were revealed with manual search of key references. After removing duplicates, 71 publications were screened and 14 relevant trials were shortlisted. Four trials were excluded due to the fact that only the protocol was reported [51], focused on alteplase induced thrombolysis [52], did not include a PCI procedure [53], or only reported the patients who received coronary artery bypass grafting instead of PCI [54]. Among the 10 remaining trials, 3 had significant resemblance in demographics of participants, authors, and institute where they were conducted [34,42,43]. Authors of these three trials were contacted multiple times to confirm if they are conducted on the same patient cohort. The consensus was to combine and weight them as one trial and to only consider those outcomes from each article which are not common among the them. Hence, only 8 trials were included in this review as shown in the flow of search strategy (Figure 1).

Geographically, the reported trials were carried out in five countries: Iran (one), Chile (two), Japan (one), China (one), Italy (two) and Canada (one) (Table 1). Total sample size of adding ten trials together was 1297 patients, a number which is derived from the subject number in the final statistics of each included trial. The mean age of the patients ranged from 56 to 68 years among nine trials with one trial not reporting the age distribution [33]. The gender ratio showed 60% to 95% male in the included ten trials. VC was administered via intravenous (i.v.) infusion in 9 trials and the oral route in one trial [55]. One trial had VC given through both i.v. infusion and intra coronary injection [39]. The dose of VC in most trials was 1 to 3 g (Table 1), with an exception of two trials from the same institute [40,44] where patients received 960 mL of 320 mmol/L of VC, equivalent to 54 g.

All trials administered VC within 12 h prior to PCI. VC was the only antioxidant regimen in all trials except 4 with additional supplements: vitamin E and beta-carotene before PCI [55], oral vitamin E [40,44] or vitamin A plus vitamin E after PCI [33] (Table 1). In one of the included trials, all patients received 325 mg of aspirin daily for entire study period [55]. Five trials enrolled patients who had ACS and underwent urgent PCI, whereas the remaining five trials included participants who had stable angina and underwent elective PCI. One trial contained patients who had either ACS or stable angina and underwent urgent or elective PCI respectively [29]. In this trial authors had assessed the outcomes of three groups: ACS patients received VC, ACS patients without VC, and stable angina patients not treated with VC. We only considered ACS patients who either or not received VC.

### 3.2. Risk of Bias Analysis

**Figure 2:**
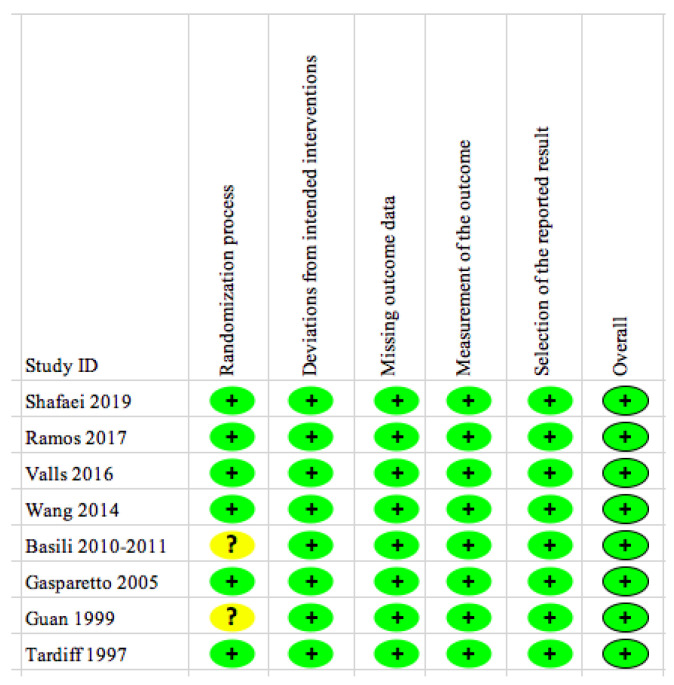
Risk of Bias of 8 Included Trials. The plus sign in green (+) shows “low risk” for bias and the question mark in yellow (?) shows “some concerns” for bias. None of the trials show “high risk” for bias.

The results of the risk of bias analysis are indicated in Figure 2. Most trials had generated random sequences for assigning intervention versus control groups. Two trials did not report randomization strategy, one of which is represented by three articles (i.e., Basili et al., 2011, Pignatelli et al., 2011 and Basili et al., 2010) [29,34,42,43], for which their corresponding authors were contacted via emails in an effort to find the details of randomization. However, we did not receive any replies. The concern about randomization did not affect the outcome values. As there was no significant difference in baseline characteristics between comparison groups, the overall risk of bias of these studies is judged as low.

### 3.3. Effect of VC Administration on Myocardial Injury

#### 3.3.1. Elevation of Troponin

An elevated level of cardiac troponins in the circulation is considered a hallmark of cardiac injury [5]. Three trials in four articles reported the blood levels of cardiac troponins within 24 h of PCI [39,41,42,43] (Table 2). Three trials analyzed cTnI using a traditional assay, whereas one trial reported the results from a high sensitivity cardiac troponin T (hs-cTnT) assay [39]. Two of the included articles had published results from same trials [42,43]. We considered the values from only one assay [43]. As a result, the findings from three trials are reported here [39,41,43].

Two trials, with a sample size of 252 and 532 respectively, showed significantly less elevation of cardiac troponins following PCI in the VC group as compared to controls [39,41] (Table 2). An additional trial with a total of 56 enrolled patients showed a trend for troponin reduction by VC treatment (*p* = 0.08) [43]. Procedure related myocardial infarction (PMI) was defined as elevation of myocardial injury marker cTnI or hs-TnT five-fold above the upper limit of normal (5xULN). The incidence of 5xULN was significantly less frequent in the VC group compared to controls (91.5% vs. 93.8%, *p* = 0.009; and 10.9% vs. 18.4%, *p* = 0.016) [39,41]. Multivariable logistic regression analysis revealed that VC treatment before PCI was an independent predictor of lower frequency of PMI (odds ratio 0.56; 95% confidence interval, 0.33–0.97; *p* = 0.037) [41]. Overall, there is evidence that administration of VC before or after PCI is associated with a decrease in blood levels of troponin and a lower frequency of PMI.

#### 3.3.2. Elevation of CK-MB

Circulating CK-MB serves as an additional biomarker of cardiac injury. Four trials presented CK-MB data as measured within 24 h after PCI [39,40,41,44]. The two trials with enrollment of 252 or 532 patients showed significantly less CK-MB elevation in the VC group compared with controls [39,41] (Table 2). One trial with a sample size of 27 patients in the VC group showed a decrease in the mean value but the statistics did not show significant difference (*p* = 0.66) [44]. Another trial did not provide the values for CK-MB, however, these investigators stated that no significant difference was noted with VC treatment [40]. The corresponding author was contacted via email twice ten days apart for retrieving the values of CK-MB without success.

The two trials that reported positive outcomes for VC reducing CK-MB levels also showed that 5xUNL of CK-MB was significantly less frequent in the VC group compared with the control group (60.8% vs. 69.9%, *p* = 0.03; and 4.2% vs. 8.6%, *p* = 0.035) [39,41]. Again, multivariable logistic regression analysis showed that administration of VC before PCI independently predicted a lower frequency of PMI as defined by CK-MB elevation (odds ratio, 0.37; 95% confidence interval, 0.14–0.99; *p* = 0.048) [41]. Consistent with the data from troponins, VC administration prior to PCI was associated with significant inhibition of CK-MB release and a lower frequency of PMI.

### 3.4. Effect of VC on Cardiac Contractility

Cardiac contractile function was measured by LVEF and TLVV. Three trials measured LVEF at 6 to 15 days after PCI [40,42,44], while one trial measured TLVV [33] (Table 2). One trial used echocardiography to assess LVEF [42], while two trials used cardiac resonance imaging (CMR) [40,44]. Two of these trials did second measurements of LVEF with CMR at 3 months after PCI with a smaller sample size (i.e., 21 and 40, respectively) than originally randomized to the treatment (i.e., 43 and 67, respectively) [40,44]. The reasons for the lower than original sample size included refusal from participants, lost to follow up, contraindication to contrast-based imaging (such as renal impairment), and death.

An improvement in LVEF due to VC, when measured at 6 to 15 days following PCI was reported in one trial [42]. The second trial showing a significant improvement in the median value for LVEF in the VC group at three months following PCI, but not at day 6 [40]. The third trial did not find any significant improvement in LVEF at 7–15 days or at three months [44]. An independent trial measured TLVV at one week or one month after PCI and found significantly lowered TLVV in the VC group, indicating better preservation of cardiac function [33]. Overall the evidence was inconclusive for the effect of VC on cardiac contractility.

### 3.5. Effect of VC on Infarct Size and Coronary Artery Restenosis

One trial used CMR to determine infarct size at two weeks for 67 participants and at three months for 43 follow-up participants [44] (Table 2). The measurements did not yield significant differences between the control and VC groups at either time point. One trial administered two pills of multivitamins to the intervention group daily for 30 days before PCI; each pill contains 250 mg of VC, 15,000 IU of beta carotene and 350 IU of vitamin E [55]. This trial assessed coronary artery restenosis at six months after PCI with coronary angiography and did not show any significant difference between the control and VC groups (38.9% vs. 40.3%, *p* = 0.89). This trial had two more intervention groups, Probucol (*n* = 58) or Probucol plus multivitamins (*n* = 56). The combination did not provide additional benefit compared to Probucol alone.

Overall the results are inconclusive for reducing infarct size, TLVV, and coronary artery restenosis, due to the fact that each of these endpoints was reported by only one study with limited numbers of patients enrolled.

### 3.6. Effect of VC on Total Antioxidant Status (TAS)

Three trials evaluated antioxidant reservoirs after PCI [33,40,44] (Table 3). The levels of ascorbate in serum, reduced glutathione (GSH) in erythrocytes, and ferric reducing ability of plasma (FRAP) were measured immediately after PCI or at 6–8 h following PCI in two trials [40,44]. Both trials showed significantly elevated ascorbate and FRAP levels at either time point in the VC group. At the time of hospital discharge (with no specific timeline provided), there was no significant difference between the comparison groups [40,44].

GSH levels were significantly higher in the VC group than in the control group immediately after PCI in one trial [44] or at 6–8 h after in two trials [40,44]. There was no significant difference between the comparison groups at the time of hospital discharge [40,44]. The third trial [33] measured total antioxidant status at 48 h and 1 month after PCI. They found significantly higher levels at 48 h (*p* < 0.01) but not 1 month following VC administration. Overall, administration of VC was associated with increased antioxidant reservoirs within 48 h after PCI.

### 3.7. Effect of VC on Reactive Oxidation Species (ROS)

Five trials in seven published articles had evaluated the impact of VC administration on ROS, measured as 8-hydroxy-2-deoxyguanosine (8-OHdG), 8-iso-prostaglandin F2a (8-iso-PGF2a), F2-isoprostanes (F2-IPs) and 8-isoprostane [29,33,34,40,41,42,43] (Table 4). The results of one trial is published in three articles [34,42,43]. Most of the trials measured blood levels of these markers except for one trial, which quantified 8-epi prostaglandin in the urine [29]. Significant reduction of 8-OHdG in the VC group was observed immediately, at 1 h [42], or 6 h after PCI [41]. Lower levels of 8-iso-PGF2a were reported due to VC at 6–8 h after PCI [43]. A similar time frame for reduction of plasma 8-isoprostane (*p* < 0.01) was also observed [40].

An assay measuring hydroperoxydes as a reflection of ROS levels in the blood showed benefit for VC (*p* < 0.05) at 48 h but not at 1 month after PCI [33]. Lack of VC association was reported by Guan et al. [29] with a urinary levels of ROS byproduct 8-epi-PGF2a (ng/mmol creatinine) measured at 0 to 150 min after PCI. The values for control group were 60 ± 8 at baseline, 81 ± 15 at 0–30 min, 93 ± 13 at 30–60 min, 122 ± 16 at 60–90 min and 82 ± 8 at 120–150 min after PCI. The values for VC group were 72 ± 12 at baseline, 98 ± 26 at 0–30 min, 104 ± 13 at 30–60 min, 123 ± 15 at 60–90 min and decreased to baseline levels at 120 to 150 min after PCI. Overall, four out of five trials showed significant association of VC with lower levels of ROS, especially when ROS products were measured in the blood within 48 h of PCI.

### 3.8. Effect of VC on Inflammation Mediators/Markers

One trial in three published articles had evaluated the effect of VC on serum levels of inflammatory markers or mediators [34,42,43] (Table 5). The inflammatory mediators or markers measured included thromboxane B2 (TxB2), soluble NOX2 derived peptide (sNOX2-dp), soluble CD40L (sCD40L), platelet CD40L, high sensitivity C-reactive protein (hs-CRP), and tumor necrosis factor alpha (TNFa).

TxB2 and sNOX2 were significantly lower in the VC group compared with the control group (Table 5) immediately or at 1 h after PCI in one trial [34]. VC administration resulted in decreases in sCD40L and platelet CD40L at these time points [42]. However, VC did not affect the level of circulating hs-CRP and TNFa as measured immediately or at 1 h after PCI [42]. Therefore, VC administration is associated with lower levels TxB2, sNOX2-dp, sCD40L, platelet CD40L, but not hs-CRP and TNFa when measured within a short time frame after PCI.

### 3.9. Effect of VC on Reperfusion Indices and Vascular Endothelial Dysfunction

Three trials determined the effect of VC on coronary reperfusion indices and vascular endothelial dysfunction [33,40,43] (Table 5). The reperfusion indices were corrected Thrombolysis in Myocardial Infarction (TIMI) Frame Counts (cTFC) and TIMI Myocardial Perfusion Grades (TMPG), both reflecting blood flow in the coronary arteries. The higher values of TMPG and lower values of cTFC indicate a better perfusion outcome. The perfusion indices (TMPG and cTFC) were surveyed immediately after PCI. TMPG 2–3 was more frequent and TMPG 0–1 was less frequent in the VC group compared with controls [40]. Similar findings were reported independently [43] with TMPG = 3 which was more frequent and TMPG < 2 which was less frequent in the VC group. The scores of cTFC were significantly reduced in the VC group [43]. These data indicate better perfusion in VC groups compared with controls.

The biomarker for endothelial dysfunction was the soluble vascular adhesion molecule (sVCAM-l). The level of sVCAM was reduced in the VC group (*p* < 0.01) at 48 h after PCI, with no significant difference noted at one month after PCI [33]. This suggests that improvement in coronary perfusion in the VC group and inhibition of endothelial dysfunction is short term.

### 3.10. Overall Results

A summary of overall results is shown in Table 6. Among the eight included trials, six trials reported positive results of VC judged by various measurements to reflect the outcomes of myocardial injury, cardiac contractility, antioxidant level, ROS, inflammation, reperfusion efficiency and endothelial dysfunction. These add to a total of 18 positive outcomes (Table 6). Among these six trials, four trials also reported certain measurements showed no statistically significant improvement with VC, including CK-MB, LVEF, infarct size, crp and TNFa. Two trials showed only negative data, with urinary oxidant measurement or coronary artery restenosis.

In total, nine types of outcomes were evaluated in the included trials seeking to evaluate possible benefit from VC administration prior to PCI. Six types of outcomes (myocardial injury, antioxidant reservoir, ROS, inflammatory mediators, coronary perfusion index, endothelial dysfunction) showed statistically significant improvement, while three types of outcomes showed inconclusive associations (infarct size, coronary artery restenosis and cardiac contractility as assessed by LVEF). By adding up the sample sizes based on significant or inconclusive associations, it was noted that 1048 participants were in the groups showing significant benefit from VC while 303 participants were in the groups with inconclusive associations.

## 4. Discussion

Significantly improved features due to intravenous (iv) administration of VC prior to PCI included lower levels of myocardial injury biomarkers (troponin, CK-MB), increased antioxidant reservoir (ascorbate, FRAP, GSH), reduced ROS (8-OHdG, 8-iso-PGF2a and 8-isoprostane), decreased inflammatory mediators (TxB2, sNOX2-dp, CD40L, and sCD40L), inhibition of vascular endothelial dysfunction (sVCAM-l) and improvement of coronary reperfusion (TMPG and cTFC). However, data is inconclusive with regard to the benefit of VC for improving cardiac function as measured by LVEF and TLVV, and reducing infarct size or coronary restenosis. The measures of LVEF from three trials showed a trend of improvement, however the statistical evidence is absent. One trial measured TLVV and showed an improvement, whereas infarct size and coronary restenosis, measured in one trial each, failed to show an effect of VC. Overall there are more trials, more outcomes and larger sample size showed the benefit of VC than that showed the lack of effect.

Half of the included trials enrolled patients with ACS whereas the other half had participants with stable angina. The trials for ACS patients administered higher doses of VC compared to those trials for stable angina patients. Both patient populations had shown significant improvements in antioxidant status, reduction of ROS, inflammatory mediators or endothelial dysfunction, and enhancement of reperfusion indices. This leads to the hypothesis that ACS may be associated with more reperfusion related oxidative and inflammatory injury compared to stable angina, and therefore requires higher doses of VC for salvage. Compared to ACS, chronic coronary syndromes such as stable angina may produce lower levels of ROS, which can be reduced by the infusion of lower doses of VC. The limitation of the included trials in cardiac injury biomarkers, such as troponin, CK-MB and LVEF, pointing to the need of future trials to demonstrate the hypothesis. A strategy for enhancing the benefit of VC is to continue administering VC for weeks to months before and after PCI procedure. Such strategy to some extent has been adopted in four of the included trials (Table 1), where the participants had either ACS or stable angina. This continuing dosing strategy may provide additional benefit in addition to the bolus dose of VC during PCI as it presumably reduces the baseline ROS and offers prolonged antioxidant protection against ROS and inflammation secondary to reperfusion injury.

The overall findings here are consistent with the data on the cardioprotective effect of VC from clinical trials of coronary reperfusion surgery patients. Two of these clinical trials showed that administration of VC before coronary artery bypass grafting (CABG) surgery significantly improved LVEF [56,57]. Similar to the PCI trials presented here, VC was given via iv infusion before CABG and was found to reduce cardiac injury [56,57]. Three systematic reviews with meta-analysis have summarized the benefit of VC for CABG and cardiac surgery patients [45,46,47,48]. The finding of improved endothelial function was documented in two reports focusing on the effect of high doses of VC on endothelial function [58,59].

### 4.1. Insights into Mechanisms

The trials showing significant benefit have used iv infusion of VC. The bioavailability following iv infusion is higher than seen with oral administration, bypassing gastrointestinal absorption and first pass metabolism. In the circulation and at the site of coronary artery, VC is readily available as a reductant, thereby reducing ROS and retarding neutrophil activation, which can produce bursts of ROS [60]. This may explain the observed reductions of ROS or inflammatory mediators. Since ROS and inflammation can cause cell death, protection against myocardial cell death is shown by decreases in PCI procedurally related troponin release.

VC might exhibit a benefit beyond reduction of ROS. Upon entering into cells, ascorbate serves as an enzyme cofactor for Fe- or Cu-oxygenases. These enzymes are important for a variety of biological functions, among which are hydroxylases for hydroxylation of proline and lysine. These reactions are not only essential for collagen synthesis but also for activation of hypoxia-induced factor-1 alpha (HIF-1a). While collagen synthesis contributes to wound repair, HIF-1a turns on many genes involved in energy metabolism, angiogenesis, and tolerance [60]. Importantly HIF-1a through coordinating gene expression attenuates proinflammatory responses [61]. These features could benefit the recovery from tissue injury due to ischemic reperfusion.

VC may also preserve the endothelial function of coronary arteries and thereby protect the myocardium. It has been shown that VC protects against endothelial damage during MI [62]. Specific actions of VC in this regard include reducing nitric oxide in the plasma, increasing vasopressor sensitivity, promoting vasodilation, and increasing micro-perfusion [63]. Ashor et al. [64] has summarized the positive effects of VC on endothelial function. Added together, VC would seem to offer cardioprotection through a combination of biological reactions, from suppressing ROS or inflammatory mediators, preserving endothelial integrity, to enhancing wound repair via collagen synthesis, and HIF-1a mediated transcriptional events.

### 4.2. Strengths and Limitations

The included trials were conducted in different geographical locations, i.e., Asia, Europe, South America and North America. Such geographical distributions provide multiethnicity in overall samples and facilitate the generalization of conclusions. The trials were either unfunded or were supported through grants from government or academic centers. None of the trials was funded by for-profit agencies, thereby reducing monetary bias. The overall risk of bias assessed with the Cochrane tool was low.

Findings from this systematic literature review have certain limitations. Four included trials had administered additional doses of VC plus alternative antioxidants, and one trial also administered aspirin to all participants for several months, all of which can potentially confound the effect of VC. The number of trials were limited and the sample sizes in most trials were small. The dose response study of VC with the desired outcomes was lacking. The studied population was relatively young with a mean age range 56 to 68 years, prohibiting extrapolation to geriatric population. There were significant heterogeneities among the reported outcomes, including the data output as means versus median, as well as discrepancies in units of measurements and distinctions in assay sensitivity. As a result, there was insufficient data for meta-analysis.

Ischemic heart disease is the number one cause of heart failure in the USA. Post MI development of heart failure is a chronic event that can take years to develop. Reduced ROS and inflammatory mediators, and improvement in coronary reperfusion efficiency in theory contribute to delay or prevention of chronic heart failure. However, the long-term course of heart failure development prohibits effective measurement of the benefit of an isolated bolus treatment with VC.

## 5. Conclusions

The qualitative synthesis of the included studies suggests that iv infusion of VC should be considered as an adjuvant therapy for PCI for prevention against reperfusion injury. Given the fact that none of the trials reviewed here has reported a detrimental effect of VC, further clinical trials with a larger enrollment could address the beneficial effects of VC, and define the therapeutic dose range of this molecule for incorporation into standard MI treatment protocols.

## Figures and Tables

**Table 1:** Demographics of 10 Randomized Clinical Trials Meeting the Inclusion and Exclusion Criteria.

	Trial	Country	Diag	Sample Size (Ctr, VC)	Age (Mean (±SD) Years		Sex (Male) *n* (%)		Vitamin C			Additional Therapy to VC Group (Yes/No)
					Ctr	VC	Ctr	VC	Route	Dose (g)	Time before PCI (min)	
1	Shafaei et al. (2019) [39]	Iran	ACS	252 (126,126)	57.18 ± 10.4	58.64 ± 10.41	97 (76.9)	104 (82.5)	IV, IC	3	0	No
2	Ramos et al. (2017) ^a^ [44]	Chile	ACS	67 (41,26)	56.16 ± 8.51	59.2 ± 11.98	NA	NA	IV	56	30	Yes ^e^
3	Valls et al. (2016) ^a^ [40]	Chile	ACS	43 (21,22)	57.1 ± 7.2	59.8 ± 13.3	19 (95.2)	20 (90.9)	IV	56	60	Yes ^e^
4	Wang et al. (2014) [41]	China	SA	532 (267,265)	58.0 ± 10.1	57.9 ± 10.4	182 (68.1)	192 (72)	IV	3	360	No
5	Basili et al. (2010–2011) ^c^	Italy	SA	56 (28,28)	68 ± 9	66 ± 8	23 (82)	24 (86)	IV	1	60	No
6	Gasparetto et al. (2005) [33]	Italy	ACS	98 (49,49)	40–86 years		74.4% Males		IV	1	60	Yes ^f^
7	Guan et al. (1999) ^d^ [29]	Japan	ACS	21 (11,10)	68 ± 4	68 ± 4	8(72)	6 (60)	IV	2	0	No
8	Tardif et al. (1997) ^b^ [55]	Canada	SA	116 (62,54)	60.3 ± 8.4	57.7 ± 11.1	61 (77)	66 (84)	PO	1	720	Yes ^g^

The trial did not have a funding source unless indicated by ^“a”^ or ^“b”^. ^“a”^ indicates funding source of Chile National Fund for Scientific and Technological Development (FONDECYT), ^“b”^ indicates funding source of the Medical Research Council of Canada. ^“c”^ indicates that three articles were combined to represent one trial, they were Basili et al., 2010 [43], Pignatelli et al., 2011 [42] and Basili et al., 2011 [34]. The trial was placebo controlled unless indicated by “^d”^. ^“d”^ indicates control group received standard treatment. ^“e”^ indicates that an oral dose of vitamin E (alpha Tocopherol) 800 IU administered before PCI, oral doses of vitamin C 500 mg/12 h and vitamin E (alpha Tocopherol) 400 IU/d administered after PCI for 84 days. ^“f”^ indicates that oral doses of vitamin C 1 g, vitamin A 50 U and vitamin E 300 mg were administered after PCI daily for one month. ^“g”^ indicates that oral doses of vitamin C 500 mg, vitamin E (alpha Tocopherol) 700 IU and beta-carotene 30,000 IU twice daily for one month before PCI and for five to seven months after PCI, plus an extra dose of Vitamin E (alpha Tocopherol) 2000 IU 12 h before PCI. SA: stable angina; ACS: acute coronary syndrome; Diag: diagnosis; Ctr: control group; VC: vitamin C group; Tx: Treatment; IV: intravenous; IC: Intra-coronary; NA: not available despite the effort of contacting the correspondent authors.

**Table 2:** Cardiac Injury and Functional Outcomes of Vitamin C Administration.

Trial [Ref]	Troponin < 24 h			CK-MB < 24 h			LVEF < 15 Days or 3 mo			Infarct Size < 15 Days or 3 mo			TLVV (mL), 7 or 30 Days		
	Ctr	VC	*p*	Ctr	VC	*p*	Ctr	VC	*p*	Ctr	VC	*p*	Ctr	VC	*p*
Shafaei et al. (2019) [39]	7.5 ng/L ^a b^	7.1 ng/L ^a b^	0.003	3.98 ng/L ^b^	3.52 ng/L ^b^	0.00									
Ramos et al. (2017) [44]				*387 u/*L *(189.0–725.0)*	*335 u/*L *(134.0–478.0)*	0.66	*49.1% ^c^* *(41.0–59.4)*	*47.3% ^c^* *(40.0–56.4)*	0.54	*21.5% ^c^ (17.0–34.2)*	*17.0% ^c^ (13.0–36.0)*	0.66			
							*47.5% ^d^* *(38.0–61.7)*	*54.6% ^d^* *(39.9–64.8)*	0.41	*19.0% ^d^ (14.0–34.0)*	*21.0% ^d^ (14.0–34.0)*	0.96			
Valls et al. (2016) [40]							*48.0% ^c b^ (56.6–33.3)*	*56% ^c b^* *(44.0–58.6)*	NS ^g^						
				NA ^g^	NA ^g^	NS ^g^	*44% ^d b^* *(34.0–56.0)*	*63% ^d b^* *(50.0–68.0)*	0.05						
Wang et al. (2014) [41]	*0.04 ng/mL* *(0.02–1.12)*	*0.03 ng/mL (0.03–0.06)*	0.02	*6.1 ng/mL (4.4–6.4)*	*4.9 ng/mL (4.1–5.7)*	0.001									
Pignatelli et al. ^h^ (2011) [42]							54.1 ± 4.7%	58.3 ± 2.9%	0.03						
Basili et al. ^h^ (2010) [43]	*△* *0.027 ng/mL* *(0.05 to 0.032)*	*△* *0.008 ng/mL* *(0.02 to 0.013)*	0.08												
Gasparetto et al. (2005) [33]													125.12 ± 29.8 ^e^	119.4 ± 29.4 ^e^	0.05
													132.0 ± 33.5 ^f^	123.4 ± 21.6 ^f^	0.05

All numbers represent means unless they are italicized, which indicate median. The data were extracted from numeric numbers from the publication unless indicated with ^“b”^, which was derived from graphs in the publication. ^“a”^ indicates high sensitivity troponin T (hs-TnT) test results. Ctr: control group; VC: vitamin C group; TLVV: telediastolic left ventricular volume. NS: non-significant; ^“c”^ or ^“d”^ indicates that LVEF or infarct size was measured within 15 days or at 3 months after PCI, respectively. ^“e”^ or ^“f”^ indicates that TLVV was measured at 7 or 30 days after PCI, respectively. ^“g”^ indicates number not available despite the effort of contacting the correspondent author; NA: not available; ^“h”^ indicates that the articles represent the same trial. △ indicates change from baseline value.

**Table 3:** Effect of Vitamin C on Antioxidant Reservoir.

Trial [Ref]	Measure		Baseline	0 h	6–8 h	48 h	1 Month	Discharge
Ramos et al. (2017) [44]	Vit C	ctr	*0.02 (0.02–0.050)*	*0.02 (0.01–0.03)*	*0.02(0.01–0.04)*			*0.03 (0.01–0.05)*
	(mmol/L)	VC	*0.04 (0.02–0.09)*	*9.63 (6.25–11.64)*	*0.72 (0.23–2.43)*			*0.02 (0.01–0.05)*
		*p*	*NS*	*<0.0001*	*<0.0001*			*NS*
	FRAP	ctr	*304.1 (213.0–429.6)*	*310.3 (227.7–400.0)*	*287 (250.2–391.2)*			*352.2 (220.0–371.8)*
	(umol/L)	VC	*271.7 (200.2–396.4)*	*8050 (50275.0–11418.0)*	*1080 (699.9–2062.0)*			*378.2 (257.9–464.3)*
		*p*	*NS*	*<0.0001*	*<0.0001*			*NS*
	GSH	ctr	*3.59 (3.21–4.54)*	*3.92 (3.26–5.28)*	*4.13 (3.48–5.06)*			*4.02 (3.34–4.43)*
	(mmol/L)	VC	*4.11 (3.61–6.56)*	*3.71 (1.75–4.60)*	*2.59 (1.61–4.15)*			*3.87 (3.55–4.86)*
		*p*	*NS*	*0.0149*	*0.0198*			*NS*
Valls et al. (2016) [40]	Vit C	ctr	0.03 ± 0.02	0.03 ± 0.04	0.03 ± 0.03			0.02 ± 0.01
	(mmol/L)	VC	0.1 ± 0.20	9.79 ± 3.87	1.79 ± 1.51			0.06 ± 0.06
		*p*	NS	<0.01	<0.01			NS
	FRAP	ctr	NA	1×	1×			NA
	(umol/L)	VC	NA	29×	4.8×			NA
		*p*	NS	<0.01	<0.01			NS
	GSH ^b^	ctr	4.2 ± 1.54	4.7 ± 2.2	4.9 ± 2.4			4.4 ± 2.0
	(mmol/L)	VC	5 ± 2.29	3 ± 2.1	2.4 ± 1.76			3.8 ± 0.7
		*p*	NS	NS	<0.01			NS
Gasparetto et at (2005) [33]	TAS	ctr	419.79 ± 34.26			526.47 ± 44.24	598.47 ± 54.99	
	(umol/L)	VC	401.95 ± 19.02			737.65 ± 51.15	647.38 ± 5 4.33	
		*p*	NS			<0.01	NS	

All numbers represent means unless they are italicized, which indicate median. Ctr: control group; VC: vitamin C group; FRAP: ferric reducing ability of plasma; TAS: Total antioxidant status; NA: not available despite the effort of contacting the correspondent authors; NS: *p* value not significant. Baseline shows the value before PCI and 0 h values were samples taken immediately after PCI. ^“b”^ indicates the number was derived from graphs in the publication.

**Table 4:** Effect of Vitamin C on ROS and Inflammatory Biomarkers.

Trial [Ref]	Measure		Baseline	0–1 h	1–2 h	6–8 h	48 h	1 Month
Valls et al. (2016) [40]	8-isoprotane ^b^ (pg/mL)	ctr	24.0 ± 16.0	28.0 ± 18.0		17.1 ± 7.0		
		VC	24.0 ± 10.4	47 ± 23.0		26 ± 13.0		
		*p*	NS	<0.05		NS		
Wang et al. (2014) [41]	8-OHdG (ng/mL)	ctr	3.8 ± 1.2			2.4 ± 1.0		
		VC	3.6 ± 1.2			4.1 ± 1.1		
		*p*	NS			<0.001		
Basili et al. (2011) ^c^ [34]	TxB2 ^b^ (ng/mL)	ctr	23 ± 2.0	26 ± 1.1	26.5 ± 4.0			
		VC	24 ± 3.5	21 ± 3.3	23 ± 3.5			
		*p*	NS	<0.05	<0.05			
	sNOX2-dp ^b^ (pg/mL)	ctr	19 ± 1.0	21.8 ± 0.2	22 ± 1.8			
		VC	21.4 ± 1.2	18.4 ± 1.4	19 ± 2.3			
		*p*	NS	0.05	<0.05			
Pignatelli et al. ^c^ (2011) [42]	8-OHdG (ng/mL)	ctr	3.7 ± 1.3	4.2 ± 1.1	4.6 ± 1.0			
		VC	3.7 ± 1.1	2.6 ± 1.1	2.7 ± 0.87			
		*p*	NS	<0.0001	<0.0001			
	hs-crp (mg/L)	ctr	*1.25 (0.80–2.00)*	*1.3 (0.75–2.0)*	1.25 (0.95–2.10)			
		VC	*1.0 (0.71–1.90)*	*1 (0.67–1.60)*	1.37 (1.0–2.0)			
		*p*	*0.451*	*NS*	NS			
	TNFα (pg/mL)	ctr	*40 (40.0–50.0)*	*40 (40.0–50.0)*	46.5 (40.0–58.0)			
		VC	*42.5 (35.0–50.0)*	*45 (40.0–60.0)*	43.5 (36.5–58.5)			
		*p*	*0.735*	*NS*	NS			
	sCD40L (ng/mL)	ctr	2.3 ± 1.2	3.2 ± 1.5	3.4 ± 1.7			
		VC	2.3 ± 1.4	2.2 ± 1.1	2.4 ± 1.0			
		*p*	0.95	0.0057	0.016			
	CD40L (MF)	ctr	4.3 ± 0.78	5.1 ± 1.3	5.4 ± 1.2			
		VC	4.2 ± 0.88	3.8 ± 1.3	3.8 ± 1.1			
		*p*	0.0724	0.0008	0.0001			
Basili et al. ^c^ (2010) [43]	8-iso-PGF2a (pg/mL)	ctr	*126 (85.0 to 170.0)*	*50 (20.7 to 102.5)*				
		VC	*142.5 (85.5 to 187.5)*	*161.5 (117.5 to 190.0)*				
		*p*	NS	<0.0001				
Gasparetto et al. (2005) [33]	ROM (U.CARR)	ctr	295.4 ± 36.30				335.6 ± 35.8	377.3 ± 39.0
		VC	308.6 ± 40.30				307.5 ± 47.1	369.1 ± 42.3
		*p*	NS				<0.05	<NS

All numbers represent means unless they are italicized, which indicate median. ^“b”^ indicates the values extracted from graphs in the publication; ^“c”^: indicates that the articles represent the same trial. 8-OHdG: 8-hydroxy-2-deoxyguanosine; TxB2: thromboxane B2; sNOX2-dp: soluble NOX2 derived peptide; hs-crp: high sensitivity C-reactive protein; sCD40L: soluble CD40L; 8-iso-PGF2a: 8-iso-prostaglandin F2 alpha; ROMs: reactive oxygen metabolites in Carratelli Units (U.CARR). 1 U.CARR = 0.08 mg % oxygen peroxide. Ctr: control group; VC: vitamin C group; NS: *p* value not significant. Baseline shows the value before PCI and 0 h values were samples taken immediately after PCI.

**Table 5:** Effect of Vitamin C on Reperfusion Indices and Vascular Endothelial Dysfunction.

Trial	Measure		Baseline	0 h	48 h	1 Month
Valls et al. (2016) [40]	TIMI (TMPG of 2–3)	ctr		79%		
		VC		95%		
		*p*		<0.01		
	TIMI (TMPG of 0–1)	ctr		21%		
		VC		5%		
		*p*		<0.01		
Basili et al. (2010) [43]	%△ cTFC	ctr	40.2	*−23%*		
	(frames/s)	VC	36.1	*−41%*		
		*p*	NS	*<0.0001*		
	TIMI (TMPG < 2)	ctr	89%	32%		
		VC	86%	4%		
		*p*	NS	<0.01		
	TIMI (TMPG = 3)	ctr		39%		
		VC		79%		
		*p*		<0.01		
Gasparetto et al. (2005) [33]	sVCAM-1	ctr	1.44 ± 0.7		2.03 ± 0.5	2.13 ± 0.8
	(ug/mL)	VC	1.53 ± 0.6		1.63 ± 0.7	1.86 ± 0.9
		*p*	NS		<0.01	NS

All numbers represent means unless they are italicized, which indicate median. TIMI: Thrombolysis in Myocardial Infarction; TMPG: TIMI Myocardial Perfusion Grade; cTFC: TIMI Frame Counts; %△ cTFC: % changes of cTFC; sVCAM-1: soluble vascular adhesion molecule 1. Baseline shows the value before PCI and 0 h values were samples taken immediately after PCI.

**Table 6:** Summary of All Outcomes.

	Studies	Trial Enrollment (Ctr, VC)	Positive Outcomes				Negative Outcomes			
			Measure	Outcome Types	Outcome Count	Enrollment	Measure	Outcome Types	Outcome Count	Enrollment
1	Shafaei et al. (2019) [39]	252 (126,126)	Troponin, CK-MB	Myocardial injury	1	252				0
2	Ramos et al. (2017) [44]	67 (41,26)	VC, FRAP, GSH	Antioxidants	1	67	CK-MB, LVEF, Infarct size	Myocardial injury, Cardiac contractility, Infarct size	3	67
3	Valls et al. (2016) [40]	43 (21,22)	LVEF, VC, FRAP, GSH, 8-isoprotane, TIMI	Cardiac contractility Antioxidants ROS, Reperfusion	4	43	CK-MB, LVEF	Myocardial injury, Cardiac contractility	2	43
4	Wang et al. (2014) [41]	532 (267,265)	Troponin, CK-MB, 8-OHdG	Myocardial injury, ROS	2	532				0
5	Basili et al. (2010–2011) ^a^	56 (28,28)	TxB2, sNOX2-dp, LVEF, 8-OHdG, sCD40L, 8-oxo-PGF2a, cTFC, TIMI	Inflammation, Cardiac contractility, ROS, ROS, Reperfusion	6	56	hs-crp, TNFa, Troponin,	Inflammation, Myocardial injury	2	56
6	Gasparetto et al. (2005) [33]	98 (49,49)	TLVV, TAS, ROS, sVCAM-1	Cardiac contractility, Antioxidant, ROS, Endothelial dysfunction	4	98				0
7	Guan et al. (1999) [29]	21 (11,10)				0	8-epi-PGF2a in urine	ROS	1	21
8	Tardif et al. (1997) [55]	116 (62,54)				0	Coronary artery restenosis	Coronary artery restenosis	1	116
	Total	1185(605, 580)			18	1048			9	303

The positive outcomes denote those showing significantly improvement in association with administration of vitamin C. The negative outcomes denote the count of those types of outcomes which are not showing significant improvement due to administration of vitamin C. ^“a”^: indicates that three articles were combined to represent one trial, they were Basili et al., 2010 [43], Pignatelli et al. 2011 [42] and Basili et al., 2011 [34].

## Supplementary Materials

The following are available online at https://www.mdpi.com/2072-6643/12/8/2199/s1: Search Strategies for multiple databases.
