# Supplementary material for: Vitamin C for Cardiac Protection during Percutaneous Coronary Intervention: A Systematic Review of Randomized Controlled Trials
Source: Nutrients. 2020 Jul 23;12(8):2199. doi: 10.3390/nu12082199 (PMC7468730; doi:10.3390/nu12082199)
Supplement: Supplementary file 1 [file nutrients-12-02199-s001.pdf]

Search Strategies for multiple databases

1. ***PubMed (NCBI)***
   Date of Search: February 18, 2020
   Number of results: 17

Search	Search Terms	Results
1	"Ascorbic Acid"[Mesh]	41905
2	Ascorbic Acid[tiab]	32420
3	Ascorbicum[tiab]	6
4	L-Ascorbic Acid[tiab]	2697
5	Vitamin C[tiab]	23156
6	Ascorbate[tiab]	16179
7	Hybrin[tiab]	2
8	Magnorbin[tiab]	0
9	OR/ 1-8	72207
10	"Angioplasty"[Mesh]	60898
11	Angioplasty[tiab]	43423
12	"Percutaneous Coronary Intervention"[Mesh]	52134
13	Percutaneous Coronary Intervention[tiab]	30932
14	Percutaneous Coronary Interventions[tiab]	3770
15	Percutaneous Coronary Revascularization[tiab]	502
16	Percutaneous Coronary Revascularizations[tiab]	4
17	OR/ 10-16	103359
18	"Myocardial Reperfusion"[Mesh]	7293
19	"Myocardial Reperfusion Injury"[Mesh]	14458
20	Coronary Reperfusion[tiab]	962
21	Coronary Reperfusions[tiab]	0
22	Myocardial Reperfusion[tiab]	1906
23	Myocardial Reperfusions[tiab]	0
24	OR/ 18-23	22109
25	9 AND 17 AND 24	17

2. ***Embase (OVID)***

   Date of Search: February 18, 2020

   Number of results: 20

Search	Search Terms	Results
1	Ascorbate.mp.	17621
2	Ascorbic Acid.mp.	89475
3	ascorbic acid/	81985
4	Ascorbicum.mp.	10
5	Hybrin.mp.	1
6	L-Ascorbic Acid.mp.	2944
7	Magnorbin.mp.	10
8	Vitamin C.mp.	24820
9	1 or 2 or 3 or 4 or 5 or 6 or 7 or 8	99736
10	Angioplasty.mp.	96445
11	angioplasty/	24673
12	Percutaneous Coronary Intervention.mp.	81183
13	percutaneous coronary intervention/	73711
14	Percutaneous Coronary Interventions.mp.	5980
15	Percutaneous Coronary Revascularization.mp.	710
16	Percutaneous Coronary Revascularizations.mp.	6
17	10 or 11 or 12 or 13 or 14 or 15 or 16	166177
18	Coronary Reperfusion.mp.	3258
19	Coronary Reperfusions.mp.	0
20	heart muscle reperfusion/	12870
21	myocardial ischemia reperfusion injury/	2944
22	Myocardial Reperfusion.mp.	3111
23	Myocardial Reperfusions.mp.	0
24	18 or 19 or 20 or 21 or 22 or 23	20260
25	9 and 17 and 24	20

3. ***Web of Science (Clarivate Analytics)***
Date of Search: February 18, 2020
Number of results: 34
Indexes: SCI-EXPANDED, SSCI, A&HCI, CPCI-S, CPCI-SSH, BKCI-S, BKCI-SSH, ESCI, CCR-EXPANDED, IC.

Search	Search Terms	Results
1	TS=(Ascorbate)	24398
2	TS=(Ascorbic Acid)	67329
3	TS=(Ascorbicum)	0
4	TS=(Hybrin)	1
5	TS=(L-Ascorbic Acid)	6031
6	TS=(Magnorbin)	1
7	TS=(Vitamin C)	91093
8	#7 OR #6 OR #5 OR #4 OR #3 OR #2 OR #1	163647
9	TS=(Angioplasty)	71556
10	TS=(Percutaneous Coronary Intervention)	50354
11	TS=(Percutaneous Coronary Interventions)	50354
12	TS=(Percutaneous Coronary Revascularization)	13055
13	TS=(Percutaneous Coronary Revascularizations)	290
14	#13 OR #12 OR #11 OR #10 OR #9	109743
15	TS=(Coronary Reperfusion)	23877
16	TS=(Coronary Reperfusions)	39
17	TS=(Myocardial Reperfusion)	41406
18	TS=(Myocardial Reperfusions)	42
19	#18 OR #17 OR #16 OR #15	44851
20	#19 AND #14 AND #8	34

4. ***CINAHL (EBSCOhost)***
   Date of Search April 9, 2020
   Number of Results: 2

Search	Search Terms	Results
1	(MH "Ascorbic Acid")	5,894
2	"Ascorbic Acid"	6,759
3	"Ascorbicum"	0
4	"L-Ascorbic Acid"	2,468
5	"Vitamin C"	5,501
6	"Ascorbate"	2,721
7	"Hybrin"	0
8	"Magnorbin"	0
9	OR/ 1-8	9,002
10	(MH "Angioplasty")	2,720
11	"Angioplasty"	19,102
12	(MH "Percutaneous Coronary Intervention")	756
13	"Percutaneous Coronary Intervention"	15,646
14	"Percutaneous Coronary Interventions"	8,207
15	"Percutaneous Coronary Revascularization"	7,585
16	"Percutaneous Coronary Revascularizations"	7,503
17	OR/ 10-16	26,678
18	(MH "Myocardial Reperfusion")	1,422
19	(MH "Myocardial Reperfusion Injury")	1,291
20	"Coronary Reperfusion"	905
21	"Coronary Reperfusions"	789
22	"Myocardial Reperfusion"	2,776
23	"Myocardial Reperfusions"	789
24	OR/ 18-23	2,867
25	9 AND 17 AND 24	2

5. ***Cochrane Library (Wiley)***
   Date of Search: February 18, 2020

Number of results: 20

Search	Search Terms	Results
1	Percutaneous Coronary Intervention	10,521
2	vitamin C	6,955
3	1 AND 2	20

6. ClinicalTrials.gov

Date of Search: April 9, 2020

Number of Results: 5

Search	Search Terms	Results
1	Condition or disease: Reperfusion	468
2	Other terms: Ascorbic Acid OR vitamin C	1259
3	1 AND 2	5
